# Supplementary material for: EGR1/GADD45α Activation by ROS of Non-Thermal Plasma Mediates Cell Death in Thyroid Carcinoma
Source: Cancers (Basel). 2021 Jan 19;13(2):351. doi: 10.3390/cancers13020351 (PMC7833439; doi:10.3390/cancers13020351)
Supplement: Supplementary file 1 [file cancers-13-00351-s001.zip › Supplemantery Materials.pdf]

Supplementary Materials

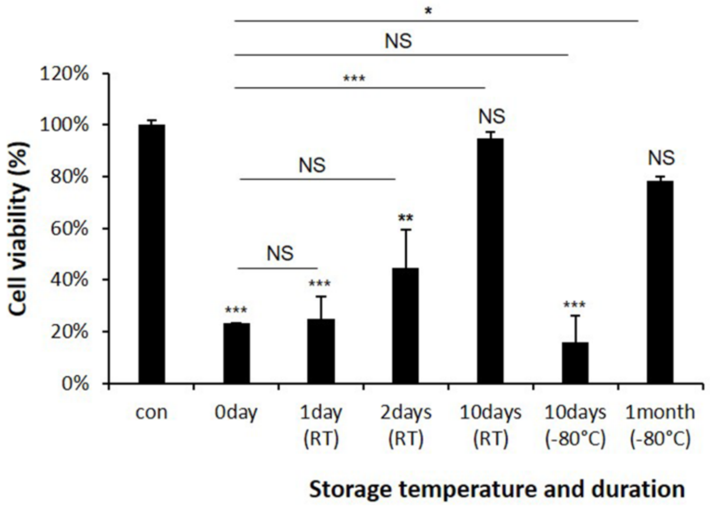

Figure S1. The effect of NTPAM according to storage temperature and duration. 2 h dose-NTPAM was stored at −80°C and room temperature (RT) for 0, 1, 2, 10 days or 1month. NTPAMs of various conditions were exposed to BCPAP cells for 24 h and cell viability was evaluated using the WST1 assay. Each figure is representative of three independent experiments. Results were analyzed using non-parametric one-way ANOVA followed by Dunnett's post-hoc test. Data were expressed as mean ± standard deviation of the mean (±SD). Differences were considered relevant at $p < 0.05$ (* $p < 0.05$, ** $p < 0.01$, *** $p < 0.001$).

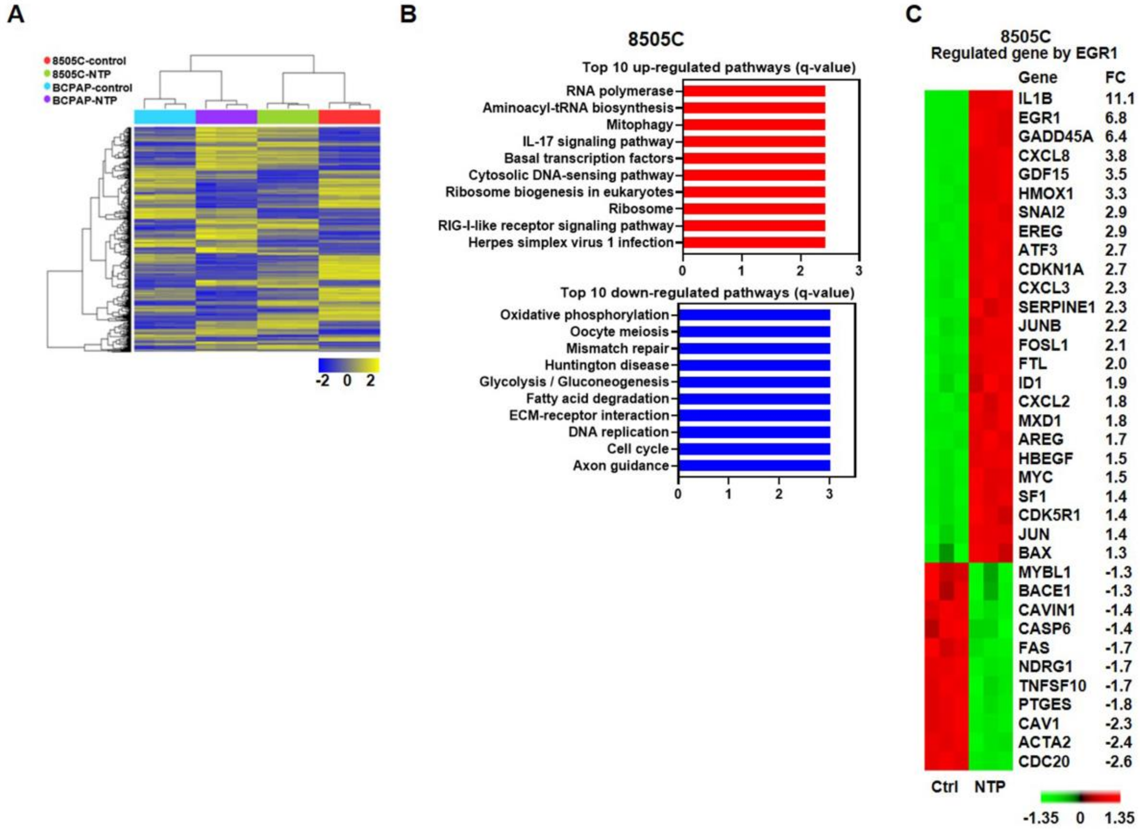

Figure S2. Transcriptomic analysis in thyroid cancer cells ± treatment with NTPAM. (A) Heatmap analysis of the significant changed genes with P value < 0.05 and Fold change > 2 comparing with or without NTPAM treatmend in BCPAP cells and 8505C cells (B) Top 10 up-regulated pathway and top 10 down-regulated pathways in 8505C cells in relation to NTPAM treatment. (C) Heatmap analysis of the significant changed genes related with EGR1 with P value < 0.05 comparing with or without NTPAM treatmend in 8505C cells.

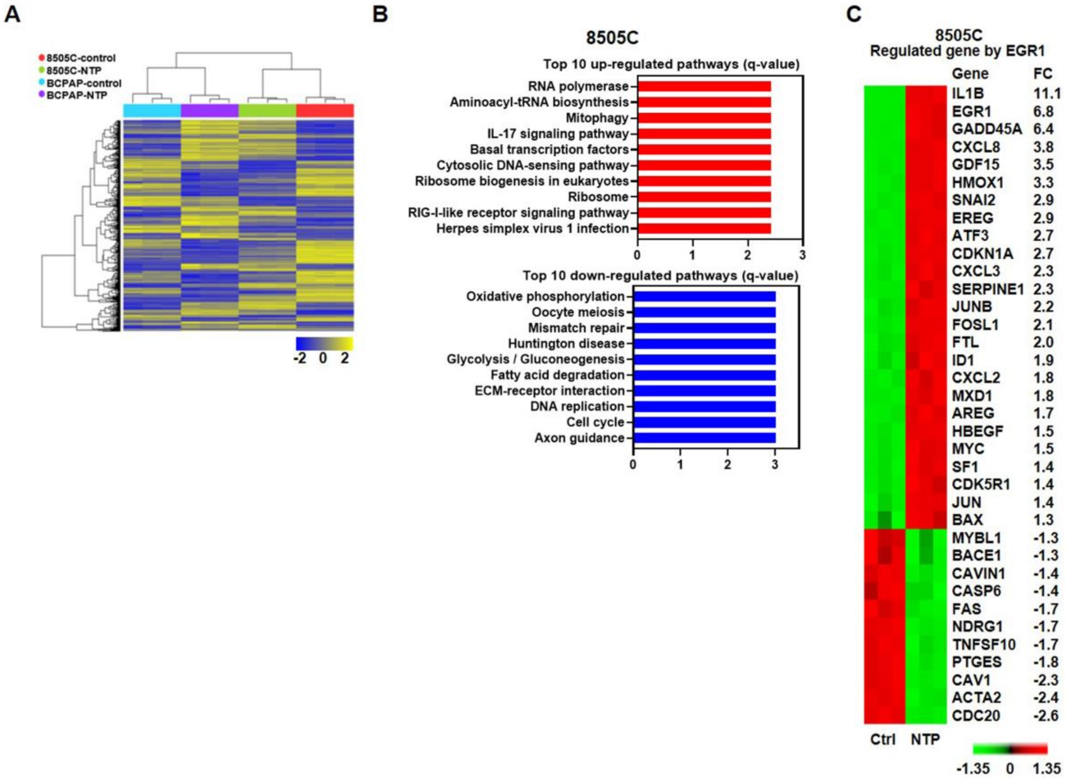

Figure S3. The effect of ATF4 in NTPAM-treated cell viability. BCPAP cells were transfected with or without ATF4-specific siRNA for 48 h, and then cells were treated with 2h dose-NTPAM for 24h. Cell viability was evaluated using the WST1 assay. Each figure is representative of three independent experiments. Results were analyzed using non-parametric one-way ANOVA followed by Dunnett's post-hoc test. Data were expressed as mean ± standard deviation of the mean (± SD). Differences were considered relevant at $p < 0.05$ (* $p < 0.05$, ** $p < 0.01$, *** $p < 0.001$).

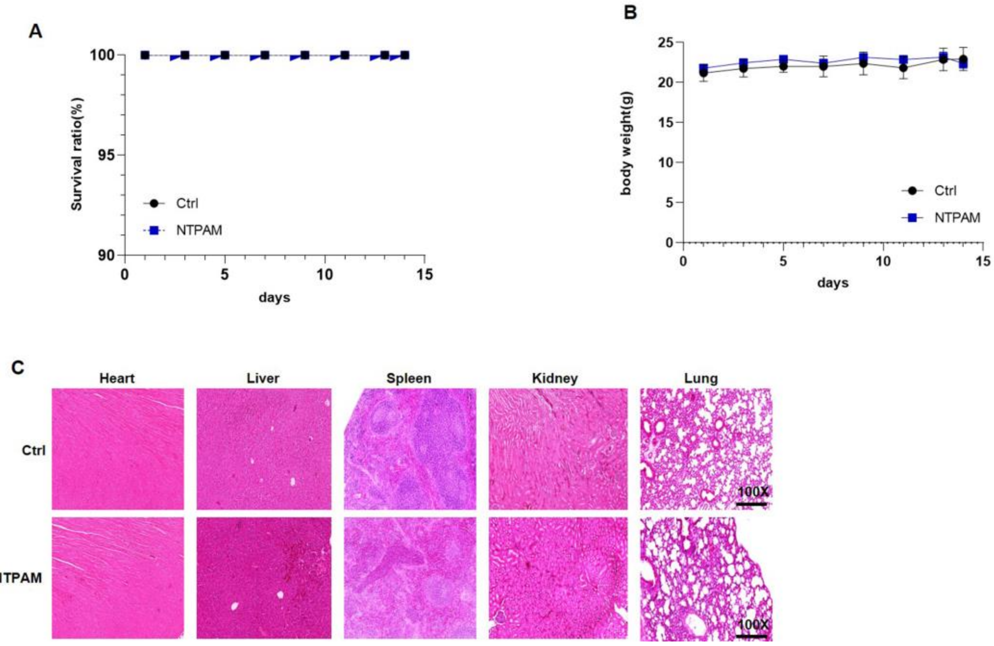

Figure S4. In vivo cytotoxic effect evaluation of NTPAM. 2 h dose-NTPAM was given to mice (C57BL/6 ,6weeks, 4mice) by introperitoneal injection (100μL) once daily for 2weeks. Survival ratio (A) and body weight (B) were monitored. C. Representative images (100X) of H&E staining of Major organs after the NTPAM treatments.

Table S1. Comparison of clinico-pathologic findings in relation to *EGR1* mRNA expression level in the THCA cohort.

Variables		Number of Patients	*EGR1* Expression		
			Low (N=250)	High (N=250)	*p* - Value
Age (years) (mean ± SD)			48.1±16.1	16.4±15.6	0.228
Gender (n, %)	Male	135	78(31.2)	57(22.8)	0.034*
	Female	365	172(68.8)	193(77.2)	
Tumor size, mm (mean ± SD)			1.65±1.19	1.30±1.04	0.014*
T stage (n, %)	T1-T2	308	162(64.8)	146(58.4)	0.141
	T3-T4	192	88(35.2)	104(41.6)	
Extrathyroidal extension (n, %)	No	348	164(65.6)	184(73.6)	0.056
	Minimal	134	73(29.2)	61(24.4)	
	Moderate/Advanced	18	13(5.2)	5(2.0)	
Lymph node metastasis (n, %)	No	277	124(49.6)	153(61.2)	0.009*
	N1a	151	91(36.4)	60(24.0)	
	N1b	72	35(14.0)	37(14.8)	
M stage (n, %)	M0	492	245(98.0)	247(98.8)	0.476
	M1	8	5(2.0)	3(1.2)	
Stage (n, %)	I	284	136(54.4)	148(59.2)	0.146
	II	52	21(8.4)	31(12.4)	
	III	111	63(25.2)	48(19.2)	
	IV	53	30(12.0)	23(9.2)	
$BRAF^{V600E}$ mutation (n, %)	No	254	119(47.6)	135(54.0)	0.288
	Yes	232	123(49.2)	109(43.6)	
	Fusion	9	4(1.6)	5(2.0)	
	Other mutation	5	4(1.6)	1(0.4)	

p values from unpaired *t*-tests for continuous parametric variables and the Mann–Whitney U-test for nonparametric variables. The chi-square test and Fisher's exact test were used to evaluate the significance of the correlations of EGR1 expression with clinical and pathological parameters.TNM classification from AJCC(American Joint Committee on Cancer) 7th edition was used. * $p < 0.05$ between the two categories for a given variable.

Table S2. Multivariable regression analysis of EGR1 expression associated with the clinic-pathologicical parameters.

Factors		Exp(β)	SE	95.0% CI	*p*-Value
Tumor size > 2cm	Age	0.9	0.006	(0.980, 1.004)	0.183
	High EGR1	1.138	0.196	(0.775, 1.670)	0.510
	Low EGR1				
LN metastasis	Age	1.015	0.006	(1.004, 1.027)	0.010*
	High EGR1	0.604	0.183	(0.422, 0.865)	0.006*
	Low EGR1				

Multivariate logistic regression analysis was performed to calculate OR and 95% CI for each covariable. The final multivariate model was based upon a stepwise method for clinical factors associated with EGR1 expression in univariate models. * $P<0.05$ between the two categories for a given variable. SE, standard error, Exp(β); OR, odds ratio, CI; confidence interval.
