# Supplementary figures and images for: EGR1/GADD45α Activation by ROS of Non-Thermal Plasma Mediates Cell Death in Thyroid Carcinoma
Source: Cancers (Basel). 2021 Jan 19;13(2):351. doi: 10.3390/cancers13020351 (PMC7833439; doi:10.3390/cancers13020351)

Figure 1.

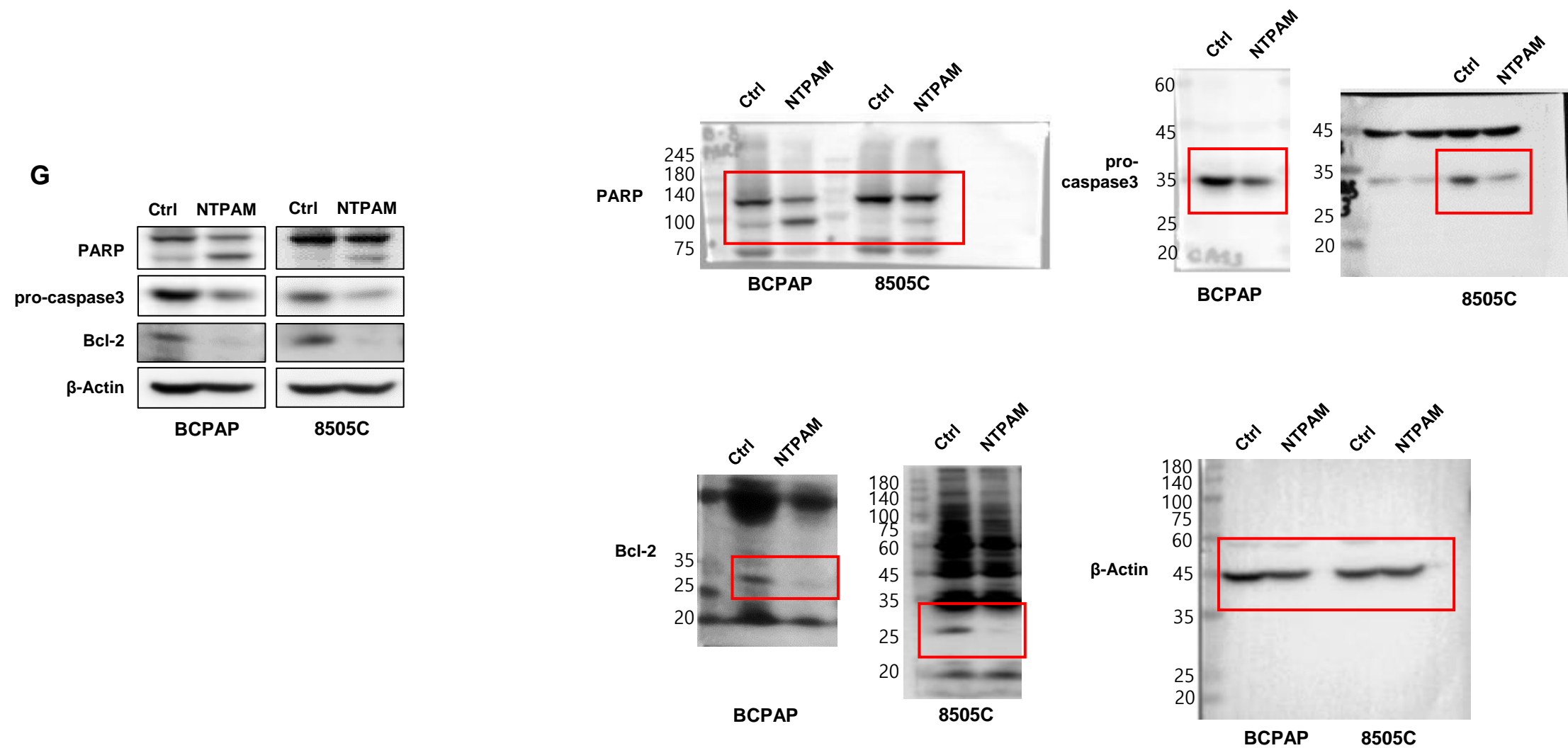

Figure 4.

C

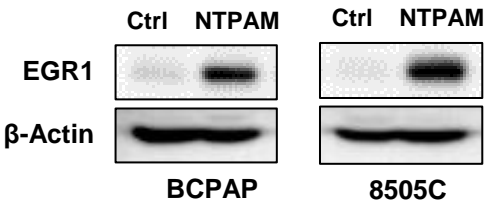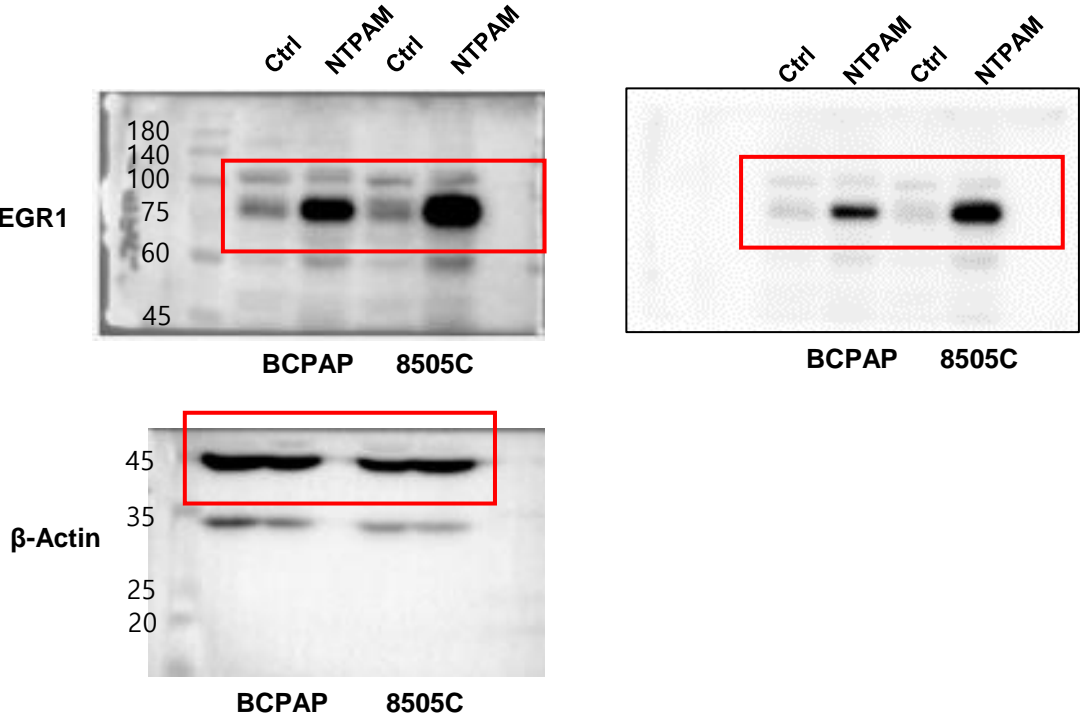

D

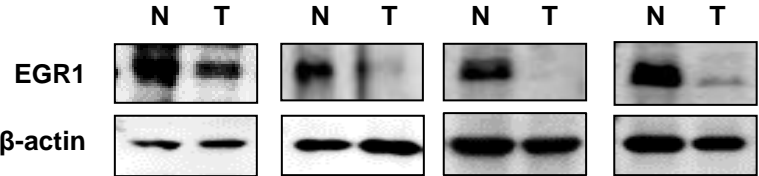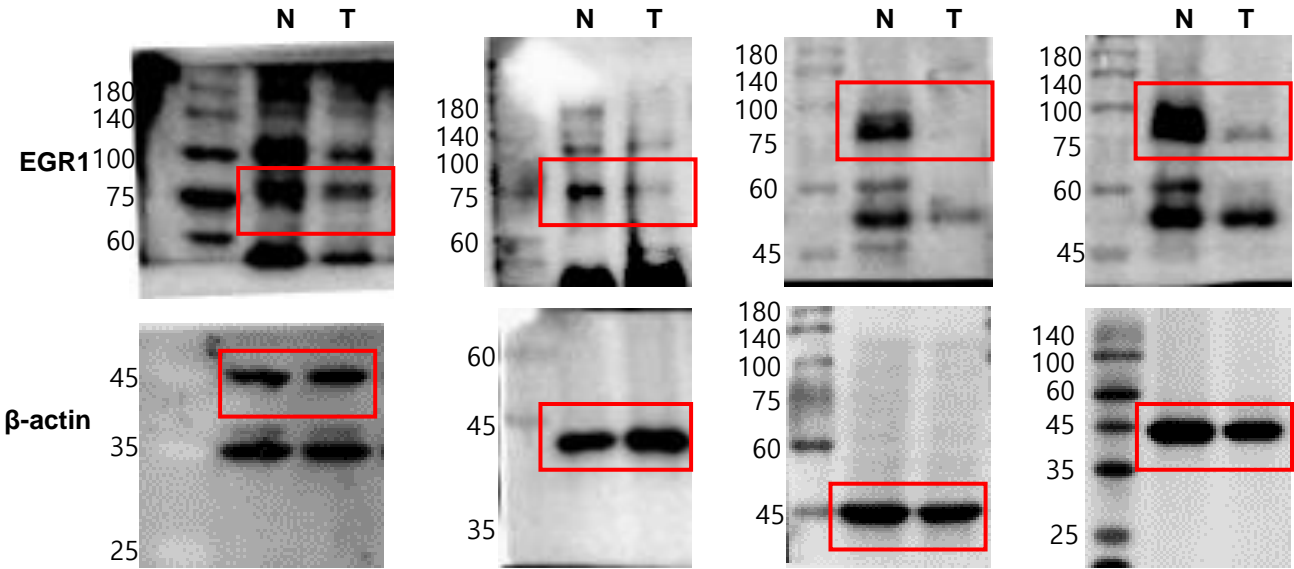

Figure 4.

E

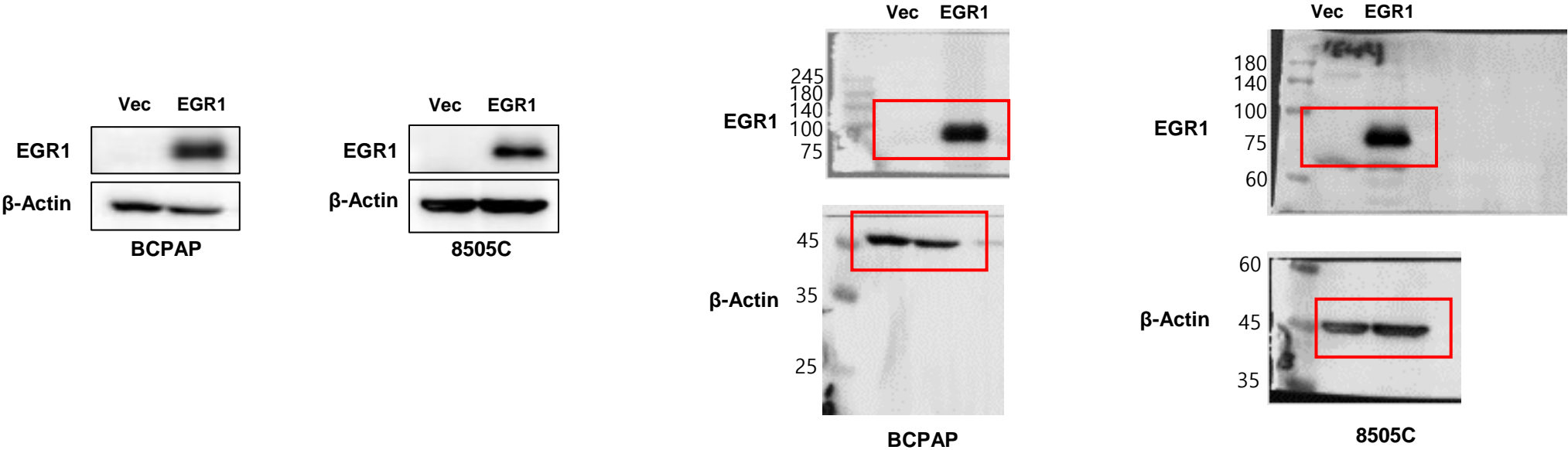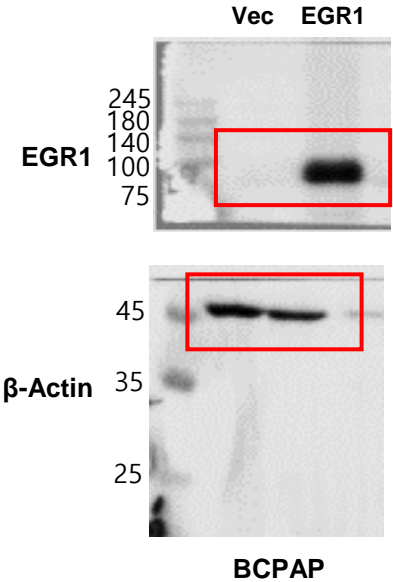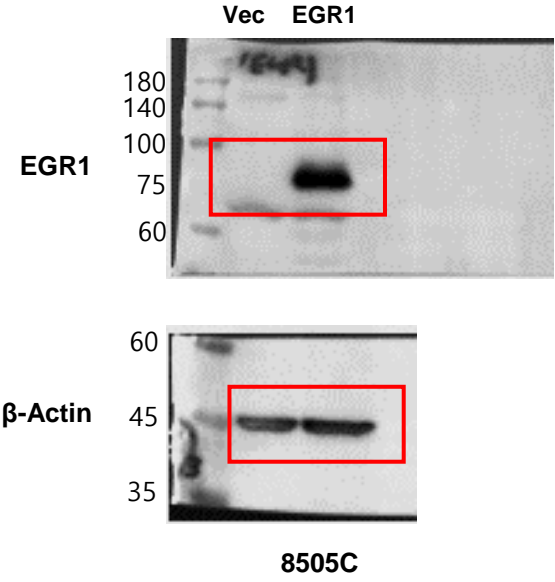

F

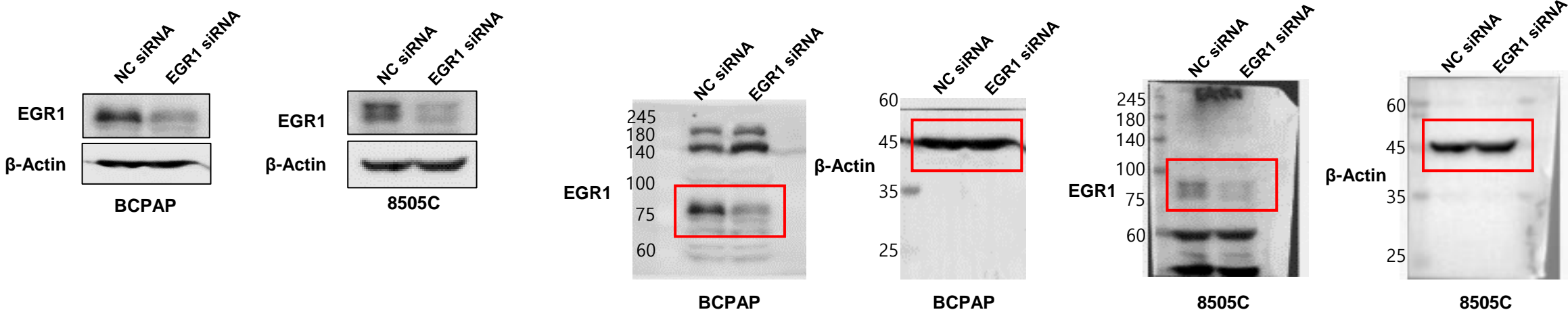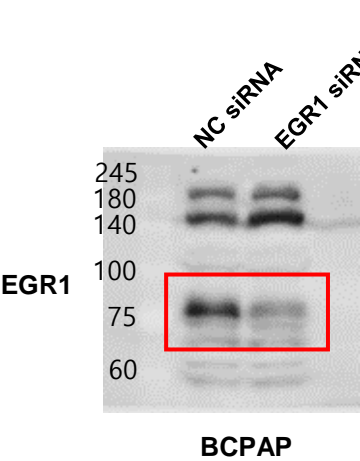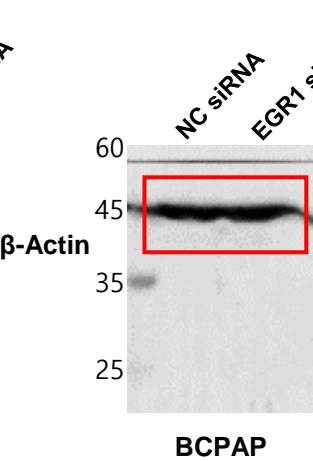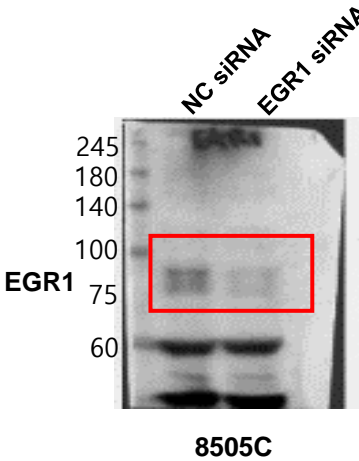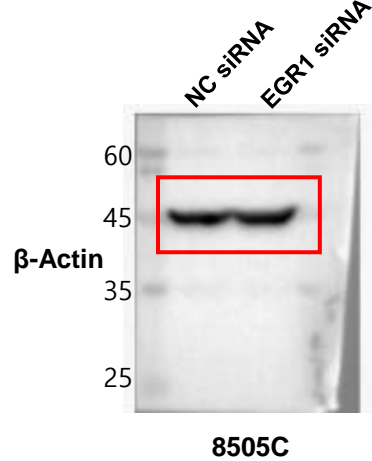

Figure 5.

C

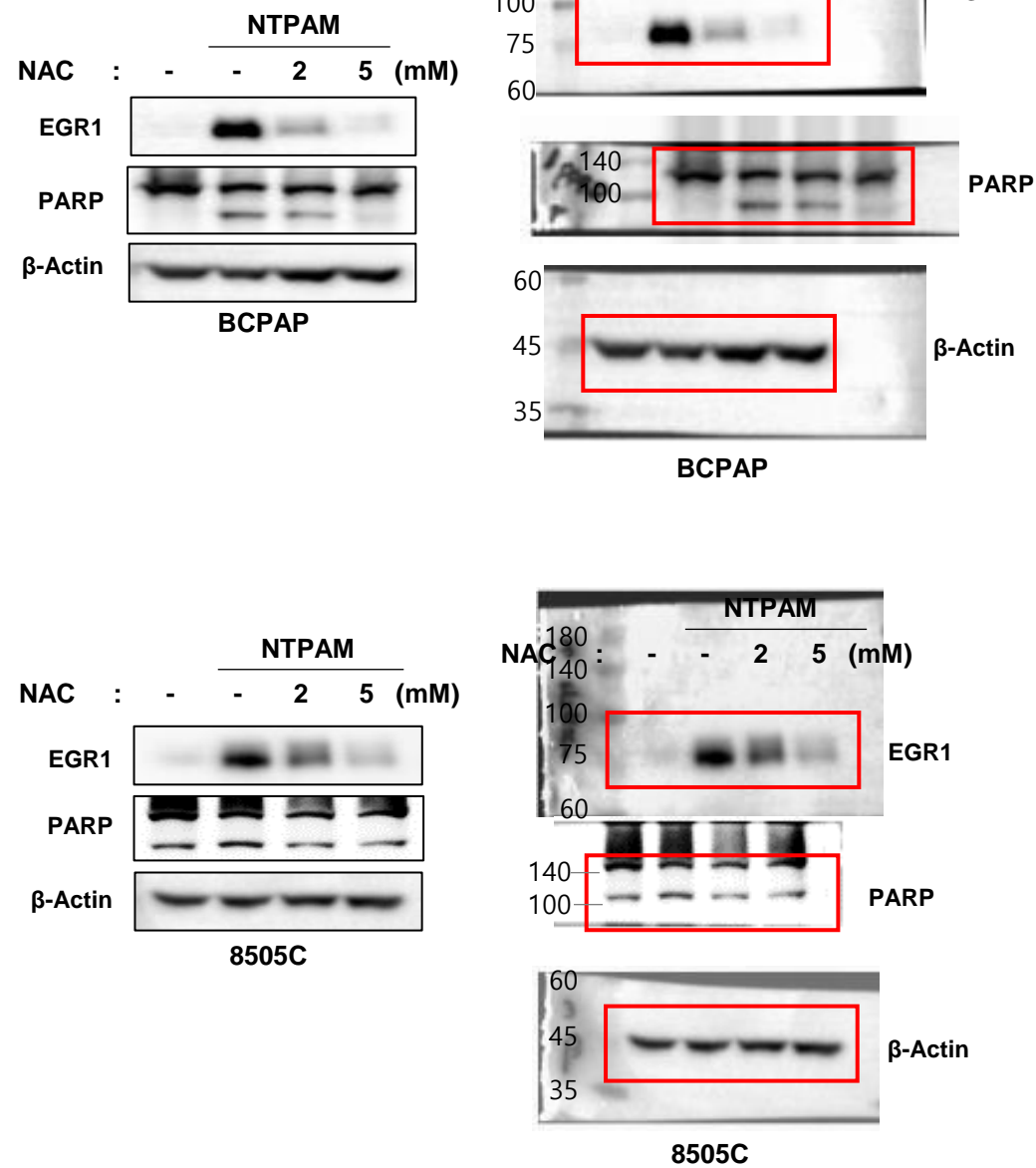

D

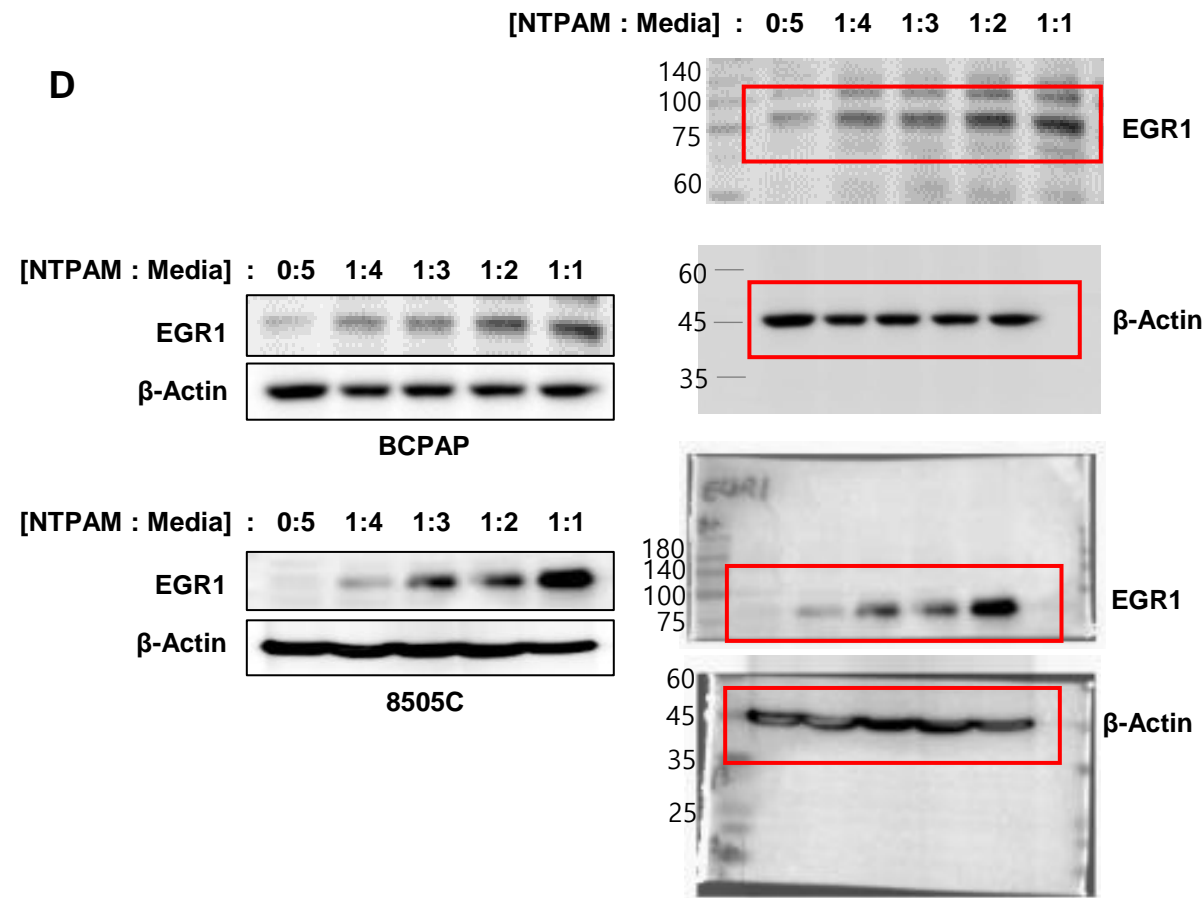

**Figure 6.**

**C**

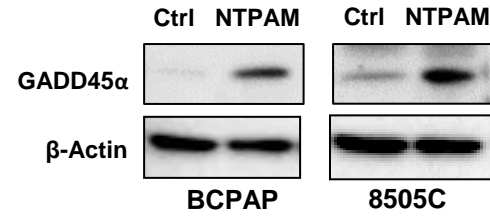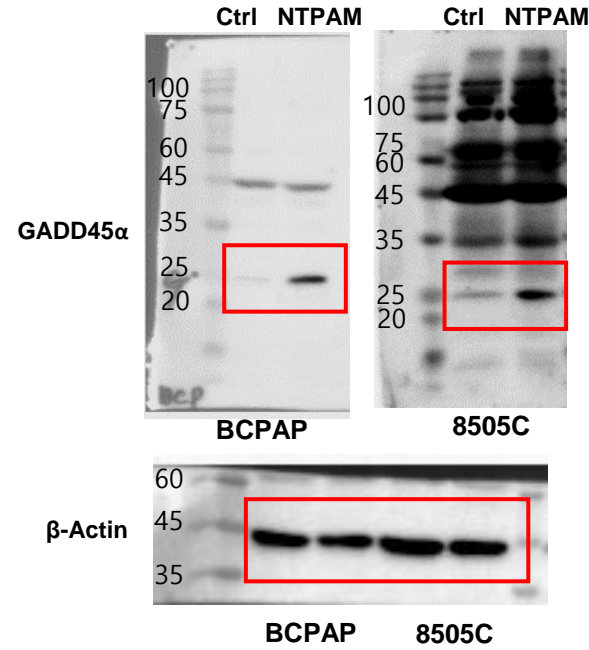

**H**

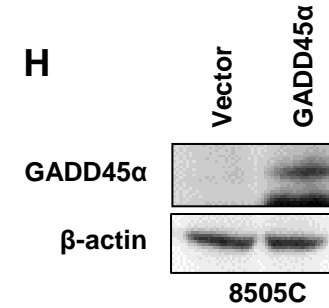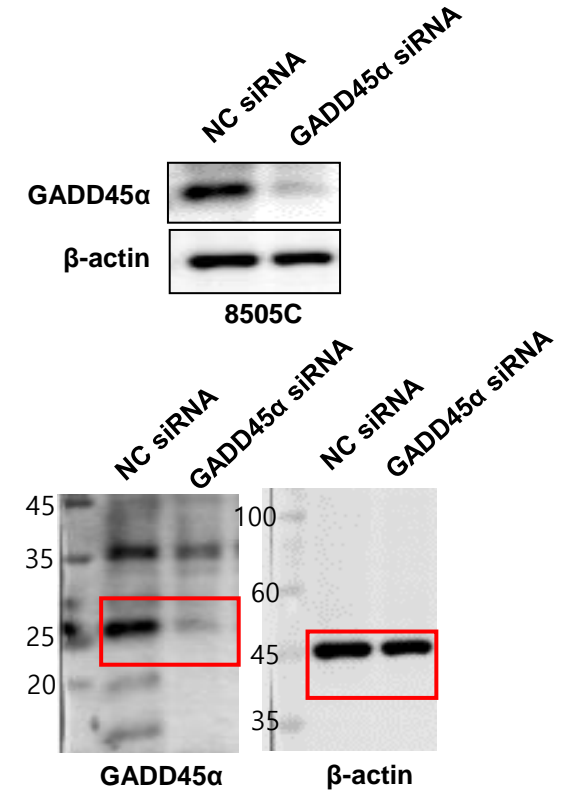

**D**

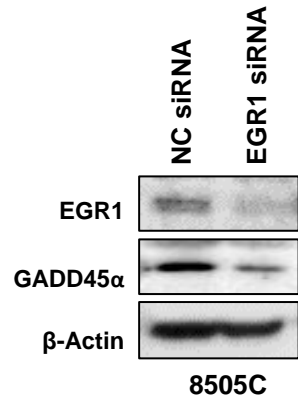

**E**

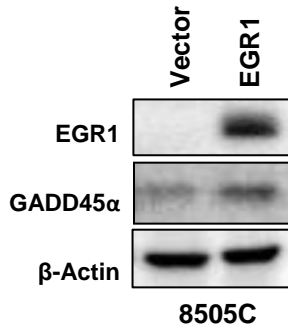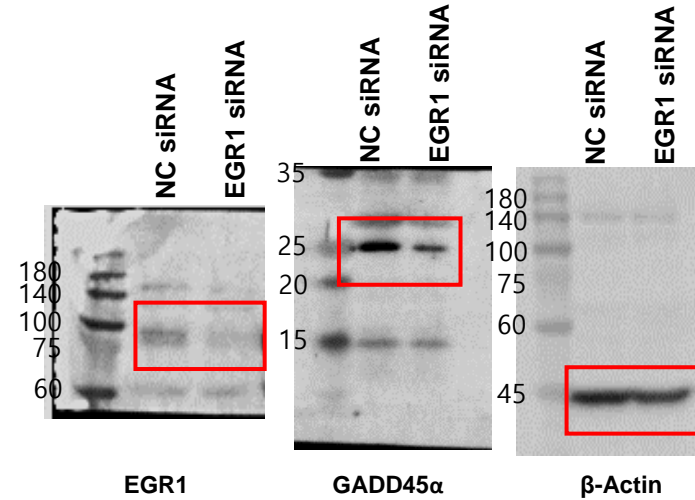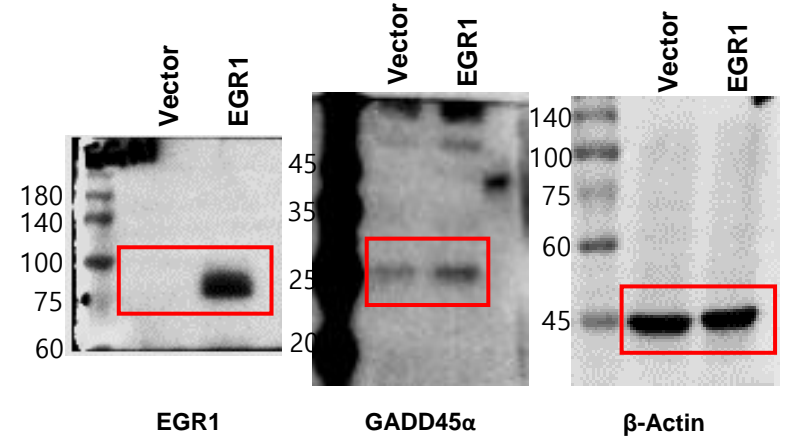

Supplement: Supplementary file 1 [file cancers-13-00351-s001.zip › original western blots.pdf]
